# Evaluating Fire Performance of Glass–Polyurethane Composite for Sustainable Cladding via Numerical and Empirical Simulation

**DOI:** 10.3390/polym15173635

**Published:** 2023-09-02

**Authors:** T. Thevega, J. A. S. C. Jayasinghe, E. Kandare, D. Robert, C. S. Bandara, L. Shi, S. Setunge

**Affiliations:** 1 School of Engineering, Royal Melbourne Institute of Technology (RMIT) University, Melbourne, VIC 3001, Australia; s3915341@student.rmit.edu.au (T.T.); everson.kandare@rmit.edu.au (E.K.); long.shi@rmit.edu.au (L.S.); sujeeva.setunge@rmit.edu.au (S.S.); 2 Department of Civil Engineering, Faculty of Engineering, University of Peradeniya, Kandy 20000, Sri Lanka; supunj@eng.pdn.ac.lk (J.A.S.C.J.); csbandara@eng.pdn.ac.lk (C.S.B.)

**Keywords:** glass–polymer composite materials, fire compliance, numerical and empirical modelling, peak heat release rate, sustainable fire safety

## Abstract

The increased demand for cladding in high-rise buildings has prompted engineers to explore alternative products utilizing recycled materials. However, ensuring fire compliance in these alternative claddings, which are predominantly composed of low-volume polymer-based composites, poses a critical challenge. Traditional experimental methods for fire evaluation are costly, time consuming, and environmentally impactful. Considering this, a numerical approach was proposed for evaluating the fire performance of glass-polymer composite materials, which contain a high proportion of recycled glass and a lower percentage of rigid polyurethane. A cone calorimeter test was simulated using Computational Fluid Dynamics (CFD) software to investigate the flammability of the novel glass–polymer composite material. This validated numerical model was employed to assess the combustibility of the glass–polyurethane composite materials and identify influential parameters using the Design of Experiments (DoE) method. Statistical analysis revealed that three material properties, namely, the heat of combustion, the absorption coefficient, and the heat of reaction, significantly influenced the peak heat release rate (pHRR) of the glass–polyurethane composite materials compared to other properties. Based on these findings, an empirical equation was proposed that demonstrates a reasonable correlation with the pHRR of low-polymer recycled glass composite materials. The outcomes of this study hold considerable importance for understanding and predicting the combustibility behaviour of low-polymer–glass composites. By providing a validated numerical model and identifying critical material properties, this research contributes to the development of sustainable fire safety solutions for buildings, enabling the use of recycled materials and reducing reliance on conventional claddings.

## 1. Introduction

The building and construction industry has witnessed an increased demand for cladding materials that must also meet fire compliance standards. However, the resources for current cladding products are finite, necessitating the exploration of innovative approaches to recover waste products and upcycle them into high-volume cladding materials. Among the abundant waste materials, glass fines show great potential for utilization in new cladding products, wherein they will replace traditional aggregate components [1]. Nevertheless, a significant challenge arises when incorporating glass fines into cladding composites, as they must be bound by combustible polymer matrices to meet structural design requirements [2,3,4,5,6,7,8]. The resulting glass–polymer composite cladding must also pass rigorous fire compliance tests specific to the building and construction industry [9,10,11,12,13,14,15]. However, the experimental evaluation of each potential formulation is both costly and time consuming. To overcome this challenge, it is imperative for engineers and researchers to develop computational models that can accurately predict the combustion behaviour of these glass–polymer composite claddings, enabling cost-effective and efficient engineering practices for assessing fire compliance.

The development of a reliable computational model for predicting the combustion behaviour of cladding materials offers numerous benefits to the building and construction industry. Firstly, accurate predictions facilitate improved fire safety by enabling designers and architects to make informed decisions regarding the selection of cladding materials, ultimately enhancing overall fire safety and potentially saving lives. Additionally, computational models help identify the most effective materials for preventing or slowing down the spread of flames, allowing designers and builders to select materials that meet fire safety requirements while minimising costs. Furthermore, the environmental impact of fires can be reduced by choosing cladding materials that are less likely to contribute to fire propagation, thus limiting the release of pollutants and greenhouse gases. By rapidly testing various cladding materials using computational models, the development cycle can be expedited, leading to the creation of more efficient and effective materials. Lastly, accurate predictions of cladding behaviour in fires provide building owners and managers with a better understanding of the associated risks, allowing them to take appropriate measures for risk mitigation.

In response to the demand for modelling tools that can accurately predict the combustion behaviour of engineering materials in real-life fire scenarios, various computational modelling approaches have been developed. A notable approach involves the use of numerical analysis and Computational Fluid Dynamics (CFD) models to assess the fire performance of fibre-reinforced polymer (FRP) composites in prefabricated modular units and various other engineering products prone to fire [4,16,17,18]. Nguyen et al. successfully demonstrated the validity of their CFD models by showing the similarities between the experimentally measured and numerically predicted fire reaction properties of organoclay/glass FRP composites [16]. Alfakhry employed computer modelling and fire simulation tools, such as Pyrosim, FDS and Smokeview, to evaluate the fire behaviour of different materials used for external cladding [19]. The objective of their study was to identify the most effective cladding material in terms of its resistance to fire propagation and ability to prevent the rapid spread of fire to upper stories. By simulating and analysing various materials, including aluminium composite panels, cement plastering, limestone, and perforated yellow bricks, researchers have aimed to provide information for the selection of cladding materials that can enhance fire safety in buildings.

Numerical simulations utilizing the CFD code Fire Dynamics Simulator (FDS) have also been used to investigate the behaviour of engineering materials in intermediate and large-scale fire tests. Incorporating Large Eddy Simulation (LES) techniques to accurately represent turbulent flows, CFD simulations focus on the performance of aluminium composite panels and polymer composite cladding in building fire scenarios [20,21,22,23]. By utilizing FDS, large-scale tests were successfully simulated, providing insights into potential improvements to materials’ fire performance without relying solely on physical experiments. These advancements in numerical modelling and simulation techniques offer valuable tools for understanding and enhancing the fire performance of engineering materials in practical applications.

This study contributes to the research field of fire engineering by reporting the accurate modelling of the combustion behaviour of the newly developed glass–polyurethane composite cladding. The developed models, both numerical and empirical, can be utilized to predict the flammability properties of other formulations containing the same constituent materials, thus reducing the need for extensive experimental testing. To achieve this, it was also crucial to determine critical parameters governing the combustion response, which can minimize the number of experiments required for input parameter collection. This study utilised the LES modelling technique in FDS to simulate the combustion behaviour of newly developed glass–polyurethane composite cladding, comprising 95% glass fines and 5% polyurethane by weight. Statistical analysis based on the Design of Experiment (DoE) approach was used to determine the sensitivity of the peak heat release rate (e.g., maximum flame intensity) to the input parameters, including the material properties. Based on the findings from the statistical analysis, an empirical equation was proposed for screening newly developed cladding materials against fire safety requirements. This empirical predictive model enables the cost-effective evaluation of the combustion behaviour of glass–polyurethane composite claddings with varying polymer and glass fines content. Ultimately, the empirical model will support the rapid screening of new cladding materials for fire-threatened infrastructure, thereby contributing to the sustainable use of recycled materials in building claddings.

The high costs associated with fire compliance tests pose significant challenges for engineers aiming to introduce recycled cladding composites into the market. Traditional experimental methods for evaluating the fire performance of cladding materials are not only costly but also time consuming and environmentally unfriendly. This study sought to address the need for a more efficient and sustainable approach by developing a comprehensive numerical and empirical modelling framework. By accurately capturing the complex interactions of heat, mass, and momentum within the material, the CFD model provides valuable insights into the fire performance of claddings. By identifying the input parameters that significantly influence flame intensity through the empirical model, we can effectively streamline and speed up the screening process for new and potential cladding materials. This approach not only reduces the cost associated with fire performance testing but also saves valuable time in the evaluation of newly developed cladding materials. The outcomes of this study carry significant implications for the industry, academia, and regulatory bodies involved in building cladding design and fire safety. By understanding the combustibility behaviour of low-polymer-volume glass composites, this research paves the way for the sustainable utilization of recycled materials in cladding applications. Our findings offer engineers and architects a more informed basis for selecting and designing fire-resistant cladding systems, promoting the adoption of environmentally friendly practices and reducing reliance on conventional and finite materials.

## 2. Experimental Methodology

### 2.1. Materials and Manufacture of Glass-Polyurethane Composite Cladding

Glass particles (or fines) used in manufacturing glass-polyurethane composite cladding were sourced from RepurposeIT (Melbourne, Australia). Glass particles with granulometric sizes of ~300 μm were obtained via the mechanical grinding of kerbside glass collections. As-received glass particles were washed under running water and oven-dried at 100 °C for 12 h. Isocyanate and polyol components from which the binder polyurethane resin was developed were supplied by Nuplex (Melbourne, Australia). Dried glass particles (95 wt.%) were compounded with isocyanate/polyol resin (5 wt.%) using a mechanical mixer operated at 100 rpm. The glass/polyurethane slurry was transferred to a 100 × 180 × 12 mm steel mould. A steel caul plate was placed above the glass/polyurethane slurry, and the mixture was subjected to an elevated temperature (160 °C) and compression pressure (6.5 MPa) moulding for 1 min [1]. The resultant glass-polyurethane composite cladding was demoulded and left to cure at room temperature (23 °C) for 7 days. Cone calorimeter specimens (100 × 100 × 9 mm in thickness) were cut from the master panel using a diamond saw. Diamond-saw-cut glass-polyurethane composite cladding specimens were dried and conditioned at room temperature over 24 h prior to the cone calorimeter and other physical and thermal tests.

### 2.2. Cone Calorimeter Evaluation of Glass-Polyurethane Composite Cladding

A cone calorimeter was used to evaluate the fire reaction properties of glass-polyurethane composite cladding according to AS 3837 [24]. Cone calorimeter specimens were wrapped in aluminium foil and placed on a steel-frame sample holder that was placed ~25 mm below the cone heater. The horizontally mounted specimen was subjected to an incident radiant heat flux of 50 kW/m^2^ representative of a moderate-intensity fire. The heat release rate (HRR) and mass loss (ML) data were recorded as a function of exposure time. The HRR data were derived from oxygen consumption rates [25]. The cone calorimeter test was stopped immediately after the burning specimen flamed out.

### 2.3. Thermal Stability and Thermal Properties of Glass-Polyurethane Composite Cladding

The thermal stability of glass-polyurethane composite cladding was evaluated using Netzsch TGA/DSC Jupiter STA 449 F5 thermogravimetric analysis (TGA) equipment (Bayern, Germany). The glass-polyurethane composite cladding was mechanically ground before ~15 mg of the powdered sample was transferred to a crucible. The crucible containing the test sample was inserted into the TGA furnace, which was continuously purged using nitrogen gas flowing at 50 mL/min. The remaining mass was measured as a function of temperature between 50 and 850 °C at a constant heating rate of 20 K/min. The reference temperature, $T_{ref}$, and pyrolysis temperature range, $T_{pyro}$, which are input parameters for the numerical fire simulation model, were derived from the TGA data. The reference temperature is defined as the temperature at which the peak mass loss rate is achieved during a TGA experiment. The pyrolysis range is defined by the onset and end point of a specific thermal degradation stage, as discussed later in Section 4.

The heat flow in the glass-polyurethane composite cladding was measured using the DSC131 Evo differential scanning calorimeter (DSC) according to the EN ISO 11357-1 standard [26]. For the DSC experiment, ~5 mg of the test material was placed in a sample holder, which was then inserted into a DSC chamber. The chamber was heated from ambient temperature (23 °C) to 300 °C at a constant heating rate of 10 K/min. The heating segment was followed by a cooling step in which the temperature was reduced from 300 °C to ~50 °C at a constant cooling rate of 10 K/min. The heat of reaction, h_r_, of the glass-polyurethane composite cladding material was derived from DSC heat flow measurement. The specific heat capacity, C_p_, was calculated via the Rule of Mixtures (RoM) utilising input parameters acquired from the literature for glass particles [27] and polyurethane [28].

The thermal conductivity, k, of the glass-polyurethane composite cladding was measured via the transient line source method using a Thermtest TLS 100 transient thermal conductivity meter equipped with a 50 mm long rock needle sensor probe employed according to ASTM D5334-14 [29]. Glass-polyurethane composite cladding specimens measuring 5 × 5 × 1 cm were used in this experiment. The sensor probe consisted of a thin heating wire and a temperature sensor, both of which were sealed in a steel tube. The heating wire applied thermal energy, while the sensor measured the sample temperature at a single point. The thermal conductivity of the glass-polyurethane composite cladding was calculated based on the heating power of the needle and the slope of the temperature–log (time) profile.

### 2.4. Absorption Coefficient of Glass-Polyurethane Composite Cladding

The absorption coefficient of the glass-polyurethane composite cladding was measured using a Fourier Transform infrared (FTIR) spectrometer equipped with an attenuated total reflectance (ATR) lens, as specified in ASTM E1252 [30]. The recorded FTIR spectrum was an average of 8 scans collected between 4000 and 400 cm^−1^ at a resolution of 2 cm^−1^. The emissivity, ε, was estimated using the RoM approach based on literature-derived values for polyurethane [31] and glass [27].

## 3. Numerical Simulation of Fire Reaction Properties of Glass-Polyurethane Composite Cladding

The use of numerical models to simulate the flammability properties of engineering materials offers a practical and more affordable alternative to the often expensive and labour-intensive process of physical measurement. The CFD simulation tool is valuable for fire analysis as it can provide accurate insights into the fire behaviour of materials with different compositions. However, not all CFD software products can simulate real fires, and some are better suited for fire analysis than others. Among the various categories of available CFD software, including Simula XFLOW, Ansys CFD, COMSOL Multiphysics, OpenFOAM, FireFOAM, CFAST, and FDS, FireFOAM, CFAST, and FDS are specifically designed for fire simulation [32,33,34,35,36]. These programs use numerical methods to solve Navier–Stokes equations that are suitable for low-speed and thermally driven flow. The CFD software focuses on smoke and heat transport and is designed to describe the evolution of fire. By developing CFD simulations, researchers can study the performance of multi-scale dimensional materials or systems under different fire loads.

In this study, a PyroSim numerical model was developed for simulating the fire reaction behaviour of glass-polyurethane composite cladding. PyroSim is an FDS-based graphical user interface software that offers advanced features for simulating complex fire scenarios. There are three different numerical approaches commonly used in CFD simulation: Direct Numerical Simulation (DNS), Reynolds-Averaged Navier–Stokes equations (RANS), and LES [37]. DNS resolves the governing equations of fluid flow, from the smallest Kolmogorov scale up to the integral scale, without using turbulent models. While DNS is useful for fundamental turbulence studies, its high computational cost limits its feasibility at larger scales. RANS, another commonly used approach, averages out the turbulence effects and solves for mean flow properties. While this approach is less computationally expensive than DNS or LES, it relies on the use of turbulence models to solve the equations, which can introduce additional uncertainty. Also, RANS models do not resolve the turbulent eddies responsible for temporal fluctuations, and they are not included in Pyrosim [38]. On the other hand, LES captures important features of the turbulent flow field, such as eddy structures, without explicitly resolving all the turbulence scales. LES reduces the range of length scales that need to be computed by filtering out small scales and replacing them with sub-grid models. This makes LES computationally less expensive than DNS while still allowing it to provide sufficient accuracy, making it a more practical tool for studying turbulent flows for wide-ranging engineering problems [39,40,41,42,43]. In summary, the choice of a specific numerical approach depends on the fire simulation needs and available computational resources. In this study, an LES-based model was used for the numerical simulation of the flammability of glass-polyurethane composite cladding.

**Figure 1:**
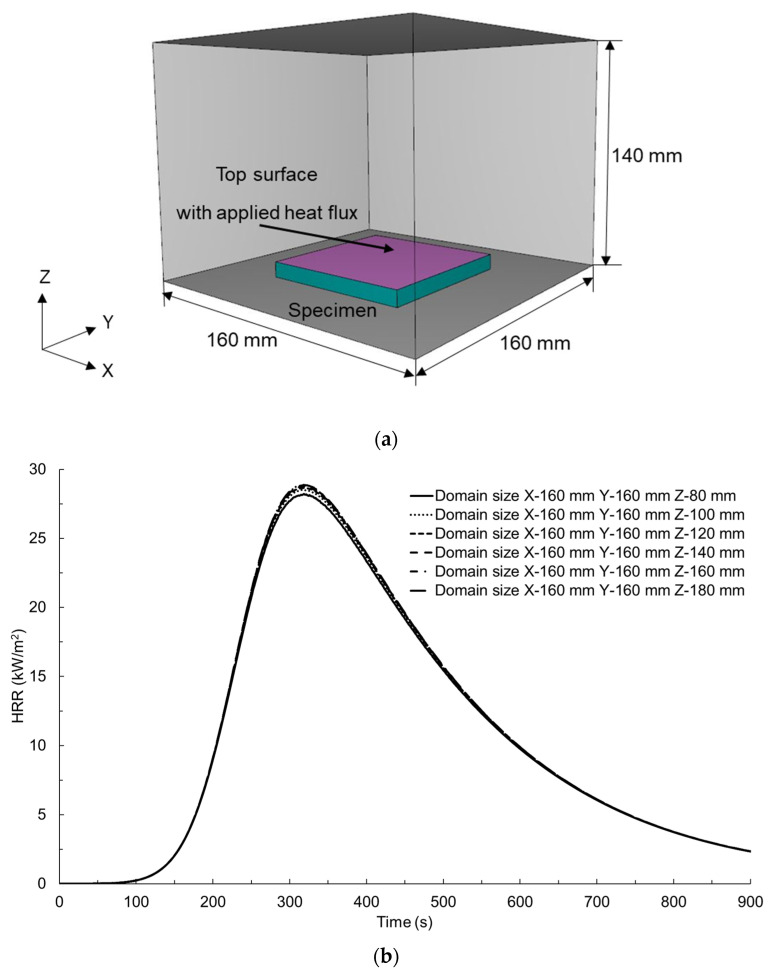
Numerical simulation model for cone calorimeter conditions in the PyroSim software. (**a**) the adopted domain size and (**b**) calculated HRR–time profiles for different domain sizes.

A numerical analysis of the fire behaviour of glass-polyurethane composite cladding subjected to an incident radiant heat flux of 50 kW/m^2^ under cone calorimeter conditions was simulated in PyroSim 2022, as illustrated in Figure 1a. The first step in setting up the numerical simulation model involved defining the domain size and grid size. In CFD simulations, both the domain and the grid sizes can impact the accuracy of the corresponding analysis. The first step involved the creation of an appropriate domain size representing the cone calorimeter specimen and thus measuring 100 × 100 × 9 mm. The X and Y dimensions were fixed at 160 mm, while the Z dimension was incrementally changed from 80 to 180 mm. The HRR–time profiles for each step change in the Z dimension are presented in Figure 1b. Convergence of the HRR–time profiles was observed for all simulated cases in which the Z dimension was at least 140 mm. As such, a domain size of 160 × 160 × 140 mm was adopted.

After an appropriate domain size was established, the next step involved the determination of a suitable grid size. Grid sizes can be determined via the integral length (IL) method or according to the turbulent resolution (TR) concept. For fire simulations, the grid size is typically selected based on the ratio of the characteristic fire diameter, $D^{*}$, to the grid cell size, δx, which is typically between 4 and 16 [21,44,45]. The characteristic fire diameter, $D^{*}$, is described in Equation (1):

(1) $$D^{*}={\left(\frac{\dot{Q}}{\rho_{\infty }c_{p}T_{\infty }\sqrt{g}}\right)}^{2/5}$$

where ($\dot{Q}$) is the fire heat release rate (kW), $\rho_{\infty }$ is the ambient temperature density of air (kg/m^3^), $c_{p}$ is the specific heat capacity of air (kJ/kgK), $T_{\infty }$ is the ambient temperature (K), and g is the acceleration due to gravity (m/s^2^). However, this approach is not suitable for scenarios in which the fire heat release rate is unknown. In such cases, the appropriate grid size can be determined by measuring the amount of resolved kinetic turbulent energy in the LES. The resolution quality is affected by the size of the fire and the grid size, which can be evaluated based on the resolved energy of the eddies for a specific grid size.

To determine the turbulent kinetic energy (TKE) in CFD simulations, one must carry out post-processing calculations involving directional velocities as in Equation (2):

(2) $$TKE=\frac{1}{2}\left({\left(\tilde{u}-\langle \tilde{u}\rangle \right)}^{2}+{\left(\tilde{v}-\langle \tilde{v}\rangle \right)}^{2}+{\left(\tilde{w}-\langle \tilde{w}\rangle \right)}^{2}\right)$$

where $\tilde{u}$, $\tilde{v,}$ and $\tilde{w}$ are turbulent velocities in the X, Y, and Z directions, respectively. The turbulent resolution is calculated according to Equation (3):

(3) $$TR=\frac{TKE}{TKE+k_{sgs}}$$

where $k_{sgs}$ is the sub-grid kinetic energy computed using Equation (4):

(4) $$k_{sgs}\approx (\frac{{\mu_{t}}^{2}}{\rho C_{v}∆})$$

where $\mu_{t}$ is the turbulent viscosity, ρ is the density of air, $C_{v}$ is Deardorff’s eddy viscosity constant, and ∆ is grid size. The accuracy of the simulation results is influenced by the resolution of the turbulent energy in the flow field, which is typically expressed as a percentage of the resolved turbulent kinetic energy with respect to the total energy. In LES, it is generally recommended to achieve a resolution of around 80% of the total kinetic energy for the accurate simulation of turbulent flows [46]. Therefore, it was crucial to select appropriate simulation parameters and grid size to achieve sufficient turbulent resolution in our LES simulations.

**Figure 2:**
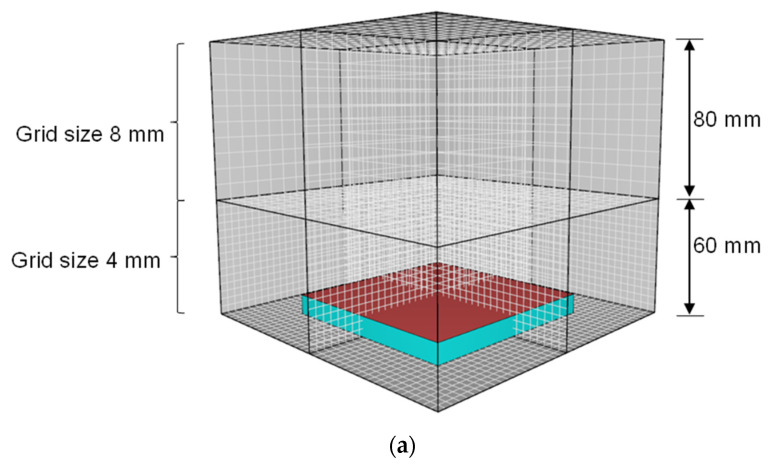

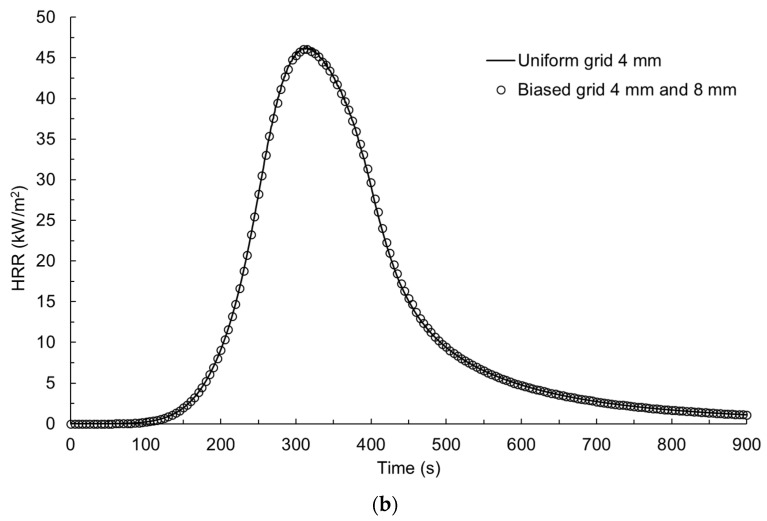
(**a**) Biased grid size modelling approach and (**b**) HRR–time profiles derived from numerical fire simulations in which uniform and biased grid size approaches were considered.

**Figure 3:**
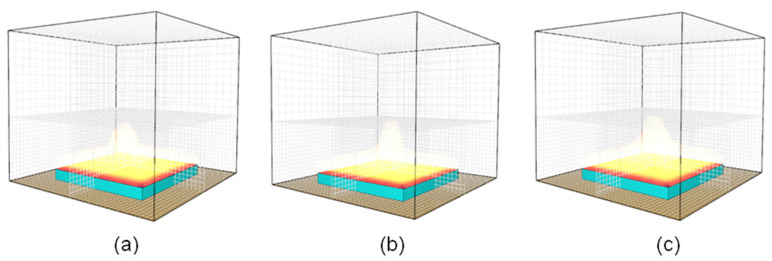
Fire development stages for a biased grid size numerical fire model simulating fire reaction behaviour under cone calorimeter conditions at (**a**) t = 265 s, (**b**) t = 315 s, and (**c**) t = 365 s.

A biased grid size approach may be implemented in CFD simulation to minimise simulation time. In this approach, the domain space is divided into sections representing fine and coarse domain spaces. It is imperative to maintain smaller grid sizes within the domain space closest to the fire initiation point (e.g., closest to the heat-exposed surface). The domain space between 0 and 60 mm along the Z dimension was modelled using a finer 4 mm grid size. The remaining domain space (e.g., 60 mm < Z < 140 mm) was modelled using a relatively coarse 8 mm grid size, as shown in Figure 2a. The HRR–time profile derived from the biased grid size approach matched the data derived from a fire simulation model in which a 4 mm grid size was used throughout the domain space, as shown in Figure 2b. The fire development stages at different times during a typical CFD analysis are shown in Figure 3. The developing fire was contained in the bottom domain space for exposure times of up to 265 s. Even after 315 s of thermal exposure, only a very small portion (<5% vol.%) of the developing fire encroached into the upper domain space. Moderate-intensity fires generally achieve peak heat release rates at exposure times less than 315 s. As such, the biased grid size approach was implemented in all numerical simulations performed in this study due to its acceptable accuracy and reduced simulation time.

Following the definition of the domain conditions, a 3D obstruction model was developed. This step was followed by the assignment of the fuel and reaction type as well as the physical and thermal properties of the test material. An incident radiant heat flux of 50 kW/m^2^ was applied, followed by the setting of the simulation time, type, and step time. All domain faces were exposed to an air atmosphere except for the bottom domain. The back surface of the simulated cone calorimeter specimen was insulated. The radiant heat flux was continuously applied to the top surface of the specimen, simulating the cone calorimeter experimental test without a specimen holder.

## 4. Results and Discussion

**Figure 4:**
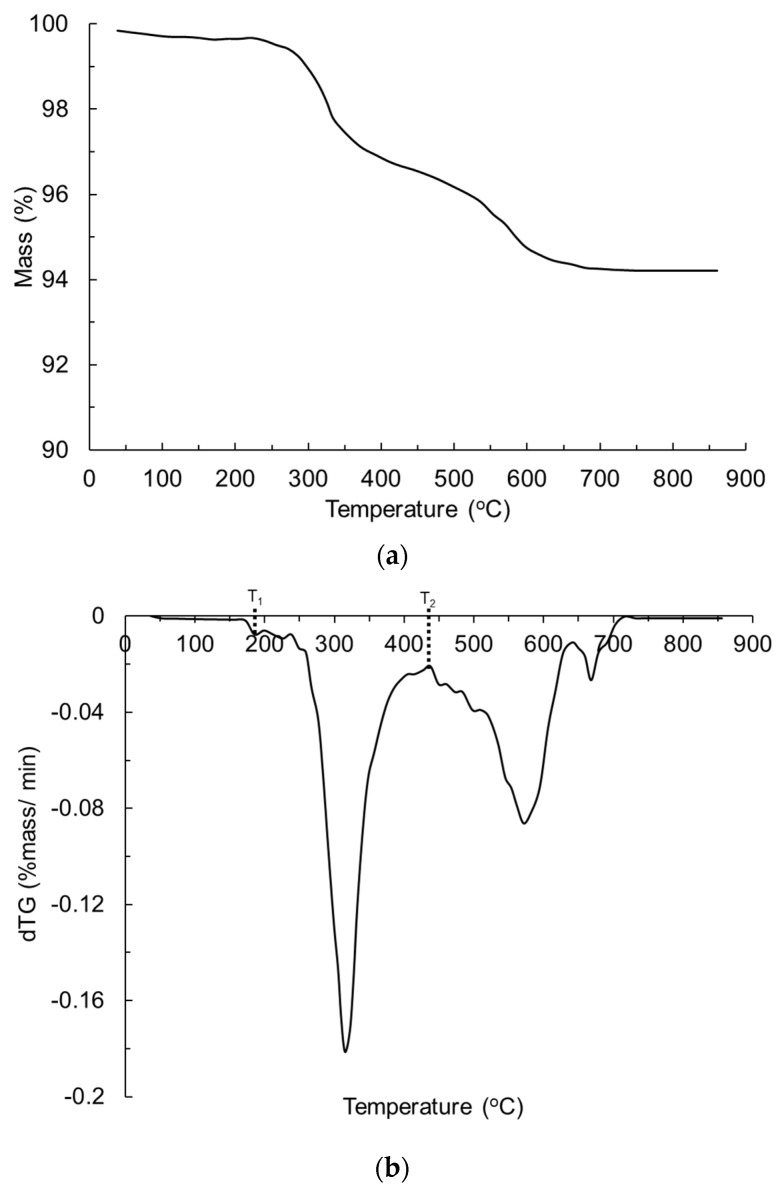
(**a**) Mass-temperature and (**b**) dTG profiles of the glass-polyurethane composite cladding.

The glass-polyurethane composite cladding density at ambient conditions was found to be ~1552 kg/m^3^. The TGA mass–temperature and corresponding derivatized thermogravimetric (dTG) profiles are shown in Figure 4. The reference temperature, $T_{ref}$, used as an input in the numerical model is the temperature at the peak mass loss rate and was recorded as 320 °C (573 K). The pyrolysis temperature range, $T_{pyro}$, calculated as the difference between temperatures $T_{2}$ and $T_{1}$ (in Figure 4), was 250 °C (523 K). The heat of the reaction, $h_{r}$, was derived from the DSC heat flow data and corresponded to 240 kJ/kg. The thermal conductivity, k, of the glass-polyurethane composite cladding material at ambient temperature was measured as 0.23 W/(mK), while the specific heat capacity, $C_{p}$, was estimated to be 0.85 kJ/(kgK). A calculated emissivity (ε) value of 0.85 and an experimentally measured absorption coefficient, α, of 10 m^−1^ were used as input parameters. The heat of combustion, $h_{c}$, measured via cone calorimetry was ~16,338 kJ/kg. The physical and thermal material properties used in the numerical fire simulation model are given in Table 1. The input parameters representing the lower (−12.5%) and upper (+12.5%) bounds relative to the nominal input values are also presented in Table 1. The lower and upper bound values were used for parametric studies, as discussed in Section 4.1.

### 4.1. Model Validation

**Figure 5:**
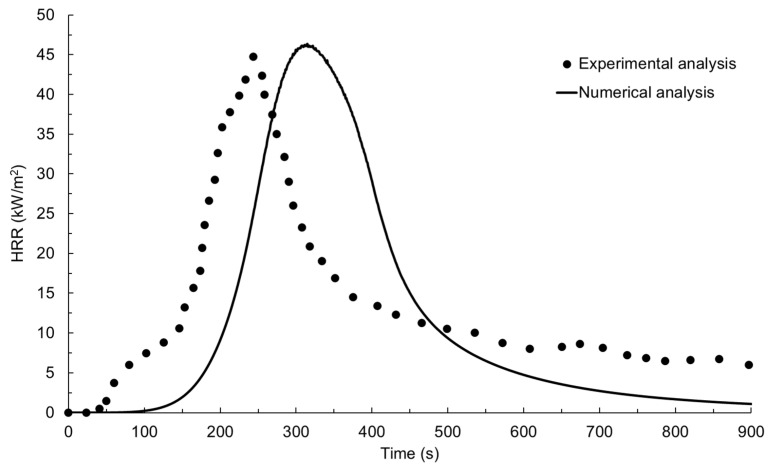
Experimentally and numerically simulated HRR–time profiles for the glass-polyurethane composite cladding.

**Figure 6:**
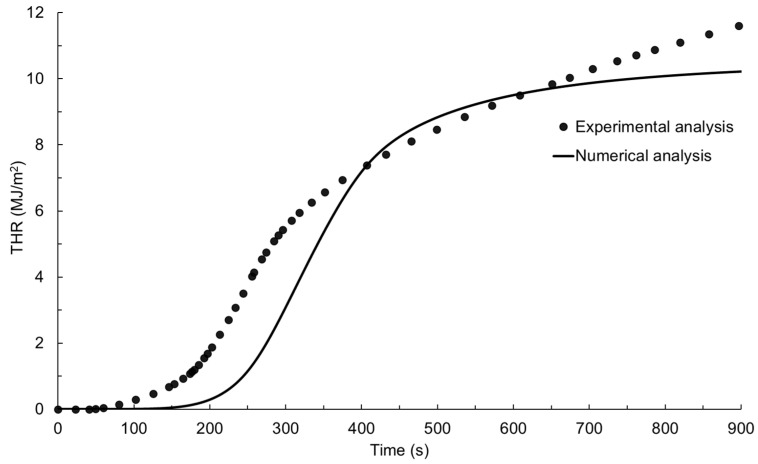
Experimentally and numerically simulated THR-time profiles for the glass-polyurethane composite cladding.

The experimentally measured HRR–time profile for the glass-polyurethane composite cladding is shown in Figure 5 together with the numerically simulated data. When exposed to an incident radiant heat flux of 50 kW/m^2^ under cone calorimeter conditions, auto-ignition occurred after 50 s of thermal exposure. The ignition of the glass-polyurethane composite cladding material generated additional thermal energy due to the combustion of volatiles released from the pyrolysis of the polyurethane binder. The combustion process led to a rapid increase in the heat release rate with a peak value (pHRR) of 45 kW/m^2^ measured after 245 s of radiant heat exposure. Due to the depletion of the polyurethane component, the HRR rapidly declined to values just above 5 kW/m^2^ a short period after the peak HRR event was achieved. The peak HRR and the time taken to reach this event are useful parameters for predicting the fire growth rate (FIGRA), which is the propensity of fire to increase in size or intensity. In this study, the fire growth rate was calculated by dividing the pHRR by the time taken to arrive at this event after ignition. FIGRA is an important parameter in fire safety engineering, as it can be used to assess the potential hazards of a fire and for the design of effective fire protection and suppression systems. For example, a high fire growth rate indicates that a fire may spread rapidly and, therefore, requires a more aggressive suppression strategy to control it. A FIGRA value of 0.23 kJ/m^2^s^2^ was determined from the experimentally measured data. Another critical parameter in the assessment of cladding material fire hazards is the total heat release (THR). The total heat release was computed by integrating the area under the HRR–time graph in Figure 5, yielding the data presented in Figure 6 and a value of 11.6 MJ/m^2^. The total heat release represents the total energy contributed by the material to a fire and is essential in assessing the fire performance of building materials such as claddings. High THR values indicate that a material is likely to release a large amount of heat energy during combustion, which can contribute to the rapid spread of fire and increase the risk of structural damage or collapse.

There was good agreement between the numerical fire simulation response and the experimentally measured data for the glass-polyurethane composite cladding, as shown in Figure 5 and Figure 6. While the numerically simulated HRR–time profile has shifted to higher temperatures, the shape of the curve resembles that of the experimentally measured data. The numerical model revealed delays in the fire development and flame out stages, indicating a potential limitation of the model in simulating these phases of fire propagation. The numerically predicted pHRR value of 46 kW/m^2^ matches the experimentally measured value of 45 kW/m^2^. The numerically derived FIGRA (0.21 kJ/m^2^s^2^) and THR (10.2 MJ/m^2^) values were within the experimental error range of the data derived from the physical experiment. The fair resemblance between the HRR–time profiles and the derived fire reaction properties indicates the high accuracy of the FDS-based fire simulation model developed in this study. Following the satisfactory validation of the numerical simulation model, we explored its sensitivity to variations in the input parameters using Minitab^®^ 2022 statistical analysis tools as discussed below.

### 4.2. Assessing the Sensitivity of the Numerical Model Results to Changes in Input Parameters

The Minitab^®^ software was used, employing the Design of Experiment (DoE) method, to perform statistical analysis of the pHRR data estimated from the numerical fire simulations of the glass-polyurethane composite cladding. The first step in the statistical analysis process involved conducting a screening analysis to identify and eliminate parameters with an insignificant effect on the pHRR. The initial screening was then followed by fractional factorial analysis, wherein parameters that had a significant influence on the pHRR values were identified. A 95% confidence level was used for the screening analysis, while factorial analysis was performed with a higher confidence level of 99%, accepting a lower error of 1%. To avoid undertaking the large number of simulations required for a full factorial analysis, a 1/8 fractional factorial approach was used, covering the maximum variation with a high confidence level. All input parameters provided to the numerical fire model were assumed to vary linearly between the lower and upper bounds and to have a significant impact on the pHRR. The input parameters and corresponding lower and upper bound levels used for parametric analysis are given in Table 1.

**Figure 7:**
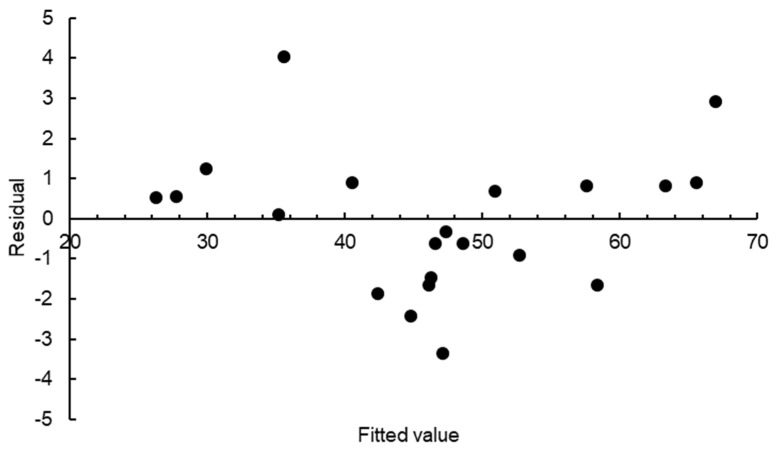
Residual plot for pHRR from screening analysis of the glass-polyurethane composite cladding.

During the screening step, twenty-one simulations were performed, with the pHRR serving as the response variable. The design matrix, normalised parameters relative to the nominal input values, and the pHRR response for the twenty-one simulations are given in Table 2. The entries −1, 0, and 1 represent the lower bound, nominal, and upper bound values for the input parameters, respectively. An analysis of variance (ANOVA) test was performed on the design matrix, and the corresponding pHRR responses are given in Table 2. ANOVA data derived from the screening stage, which involved twenty-one simulations, are presented in Table 3. The data indicate that the specific heat capacity (*p* = 0.001), emissivity (*p* = 0.022), absorption coefficient (*p* = 0.000), heat of combustion (*p* = 0.000), reference temperature (*p* = 0.001), pyrolysis range (*p* = 0.000), and the heat of reaction (*p* = 0.000) all had a significant influence on the pHRR of the glass-polyurethane composite cladding material when the significance level was set at 5%. Density and thermal conductivity were found to have insignificant effects on the pHRR response. The screening analysis demonstrated a high R^2^ value of 98% and an Adjusted R^2^ value of 97%, indicating a significant amount of variation in the response function. This is an indication that the model is a good fit for the data and that the selected independent variables are important in predicting the dependent variable. The residual plot, which is shown with an even balance of pHRR response values on either side of the zero residual line in Figure 7, is a good indication of the reliability and validity of the statistical analysis conducted.

Two input parameters that had a negligible impact on the pHRR response were eliminated before a detailed analysis was performed using the fractional factorial method. The fractional factorial analysis assessed the significance of the seven parameters on the pHRR of the glass-polyurethane composite cladding based on sixteen runs. Table 4 presents the design matrix and the pHRR response derived from the fractional factorial analysis.

**Figure 8:**
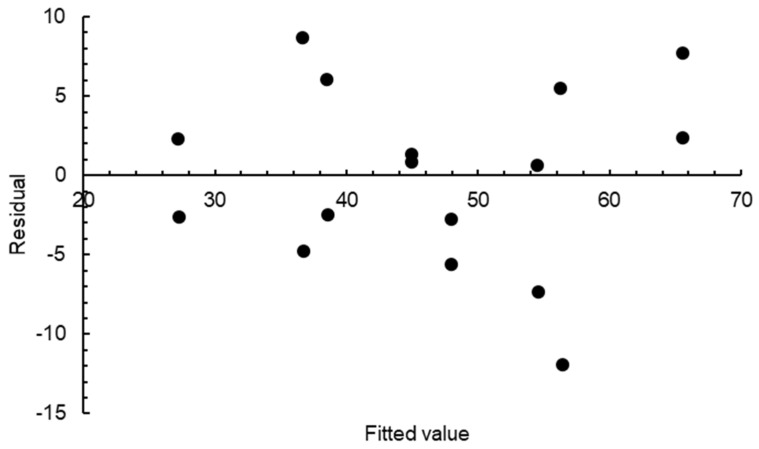
Residual plot for pHRR from fractional factorial analysis.

To maintain brevity, ANOVA data derived from the fractional factorial assessment are not presented here. Instead, only those parameters found to have significant effects on the pHRR value are discussed. The heat of combustion (*p* = 0.000), absorption coefficient (*p* = 0.010), and heat of reaction (*p* = 0.004) were found to have significant effects on the pHRR of the glass-polyurethane composite cladding material at a 1% significance level. The R^2^ (82%) and adjusted R^2^ (77%) values were within an acceptable range, indicating a strong correlation between the independent variables and the dependent variable and the development of a highly effective model for explaining the variation in the response function. The residual plot of each factor on the pHRR is shown in Figure 8 based on the fractional factorial analyses, and, again, an even balance of pHRR response values on either side of the zero residual line is a good indication of the reliability and validity of the statistical analysis conducted.

**Figure 9:**
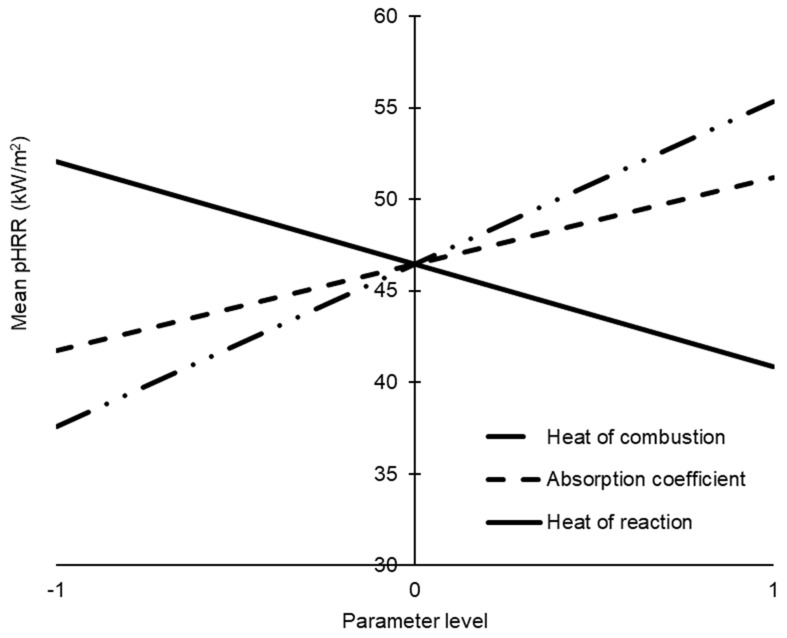
Variation in the pHRR response with changes in the heat of combustion, absorption coefficient, and heat of reaction of the glass-polyurethane composite cladding.

It was crucial for us to identify the key parameters with significant effects on the pHRR in order to reduce the complexity of further analysis. In this case, the fractional factorial analysis led to the identification of three critical parameters that had a significant influence on the pHRR of the glass-polyurethane composite material: the heat of reaction, the heat of combustion, and the absorption coefficient. These parameters should be carefully considered during material selection to ensure the resulting cladding material satisfies the desired level of fire performance. The effects of the three critical input parameters on the pHRR value were assessed by varying their magnitudes between the lower and upper bounds. The pHRR value was selected for this analysis since it provides valuable information on flammability and potential fire hazards in the event of a fire. The pHRR value provides an indication of flame intensity and the extent to which a developing fire can spread. As shown in Figure 9, when a linear variation in the input parameters was assumed, the pHRR monotonically increased with the increase in the heat of combustion and the absorption coefficient. In contrast, the increase in the heat of the reaction led to lower pHRR values.

Since the heat of combustion is the amount of heat released when a substance undergoes complete combustion with oxygen, it is conceivable that the HRR values should increase with the increase in the heat of combustion. Materials with high absorption coefficients are more effective at absorbing radiant energy, which, in turn, accelerates pyrolysis and increases pHRR values. When the heat required to break down the polymer binder during pyrolysis is high, reduced amounts of combustion volatiles are produced, resulting in lower pHRR values. That is, when creating new glass-polymer composite cladding formulations, attention should be paid to the heat of combustion of polymer binders. Polymers with relatively high heat of combustion values should be avoided. On the other hand, thermally stable polymers (e.g., polymers with an elevated heat of reaction) are more suitable since the design objective for cladding material is to minimise the fire growth rate and flame intensity. Further, the amount of polymer and the resultant absorption coefficient of the glass-polymer composite are crucial parameters that should be carefully considered to meet stringent fire compliance requirements for cladding applications.

### 4.3. Estimating Maximum Flame Intensity Using an Empirical Model

Numerical fire simulation models offer a cost-effective, accurate, and quicker alternative to performing physical experiments in relation to assessing the fire performance of new cladding materials. Further, numerical models can simulate complex and detailed physical processes and are easily adaptable to different scenarios and conditions. Despite a suite of advantages of numerical models over experimental data acquisition, there is room to further simplify predictive fire simulation models to reduce computational costs and time. Empirical models are generally simpler and easier to understand than numerical models, as they are based on direct relationships between input and output variables. Empirical models are often computationally quicker than their numerical counterparts. If the data used to develop the model are representative of the conditions of interest and the empirical model has been validated, this approach can provide quick, approximate predictions for a range of conditions. A linear empirical equation (Equation (5)) describing the relationship between the three significant parameters and the pHRR was developed using a multivariable data-fitting method based on the linear regression model.

(5) $$\mathrm{pHRR}=(4.4\times 10^{-3}h_{c})+(3.8\alpha )-(2.0\times 10^{-1}\;h_{r})-17$$

**Figure 10:**
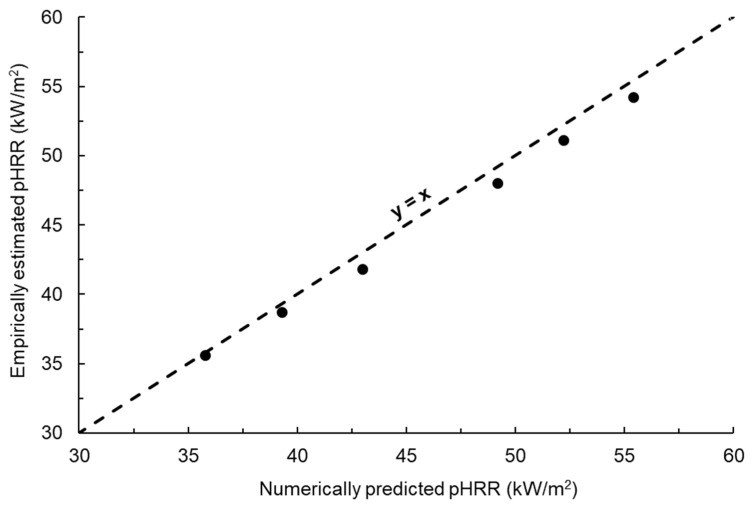
Comparison between the pHRR values predicted using the numerical fire model and those estimated from the empirical equation for the glass-polyurethane composite cladding.

The numerical fire model was used to simulate six different scenarios with three significant input parameters varied simultaneously. The same parameters were also used as an input for the empirical model. The pHRR values estimated using the numerical and empirical models were compared. The results showed strong agreement between the data estimated using the two models, with the data points closely following the x = y line, as shown in Figure 10. Although numerical models are more suitable for detailed fire scenario analysis and investigating underlying mechanisms compared to empirical models, this study demonstrates the potential of empirical models for the initial fire performance screening of new cladding materials. The empirical model presented herein can be used for the rapid screening of different cladding formulations such as with respect to changes in the type and weight percent of the polymer binder. This study validates the feasibility of using both numerical and empirical models to predict the maximum flame intensity (pHRR) in the event of glass-polyurethane composite cladding catching fire.

## 5. Conclusions

In this study, the fire performance of a new cladding composite material incorporating recycled glass particles and polyurethane as a binder was assessed using a combination of experimental, numerical, and empirical methods. The findings of this study have several important implications and provide insights into the combustion behaviour of glass-polymer composite materials. The significance of this study lies in the use of numerical simulations as a cost-effective and environmentally friendly alternative to traditional experimental tests. The validated numerical model allowed us to conduct a parametric study, which identified the key material parameters that significantly influence the peak heat release rate (pHRR), a critical indicator of thermal performance. By incorporating these influential parameters, including the heat of combustion, absorption coefficient, and heat of reaction, an empirical equation was proposed to evaluate the combustion behaviour of glass-polyurethane composite materials in terms of pHRR.

The application of numerical simulations and the development of the empirical equation offer valuable benefits for researchers and practitioners in the field. They enable a more efficient assessment of material performance, saving time and resources while providing insights into the thermal behaviour of newly developed materials during a fire. This knowledge allows individuals to make informed decisions regarding the suitability of glass-polymer composite materials for specific applications, thereby enhancing fire safety in the building and construction industry. However, it is important to acknowledge the limitations of this study. The accuracy of the proposed empirical equation is contingent upon the range of parameters investigated. Therefore, future research should explore a wider range of glass and polymer proportions to enhance the precision and applicability of this equation across different compositions.

In conclusion, this study contributes to the understanding of the thermal behaviour of glass–polymer composite materials and their suitability for building claddings. The combination of numerical simulations, parametric studies, and the development of an empirical equation provides a valuable tool for evaluating combustion behaviour and improving fire safety in construction. Future research should focus on expanding the parameter range to further refine the proposed equation and advance the use of recycled glass–polymer composites in building materials.

## Figures and Tables

**Table 1:** Physical and thermal input parameters for the numerical fire simulation model. Lower- and upper-bound values are 12.5% lower and greater, respectively, than nominal input values.

Property	Source	Lower Bound	Nominal Value	Upper Bound
$\mathrm{Density}\;\rho_{\infty }$ (kg/m^3^)	Direct measurement	1358	1552	1746
$\mathrm{Reference}\;\mathrm{temperature},\;T_{ref}$ (°C)	TGA	280	320	360
$\mathrm{Pyrolysis}\;\mathrm{range},\;T_{pyro}$ (°C)	TGA	219	250	281
$\mathrm{Heat}\;\mathrm{of}\;\mathrm{reaction},\;h_{r}$ (kJ/kg)	DSC	210	240	270
Thermal conductivity, k (W/mK)	Transient line source	0.20	0.23	0.26
$\mathrm{Specific}\;\mathrm{heat}\;\mathrm{capacity},\;C_{p}$ (kJ/kgK)	Rule of Mixture method	0.74	0.85	0.96
Emissivity, ε (−)	Rule of Mixture method	0.74	0.85	0.96
Absorption coefficient, α (m^−1^)	FTIR	8.75	10.00	11.25
$\mathrm{Heat}\;\mathrm{of}\;\mathrm{combustion},\;h_{c}$ (kJ/kg)	Cone calorimeter	14,296	16,338	18,380

**Table 2:** Design matrix and pHRR responses for twenty-one screening analysis runs.

Run	$\rho_{\infty }$	$T_{ref}$	$T_{pyro}$	$h_{r}$	k	$C_{p}$	ε	α	$h_{c}$	pHRR
1	1	1	−1	1	−1	−1	0	−1	1	44.74
2	−1	1	1	0	1	−1	−1	−1	−1	28.23
3	−1	1	−1	−1	−1	1	−1	1	0	51.81
4	1	0	1	−1	−1	1	1	−1	−1	35.19
5	1	−1	1	−1	0	−1	−1	1	1	70.03
6	−1	1	1	−1	−1	−1	1	0	1	58.49
7	0	1	1	1	1	1	1	1	1	47.10
8	−1	−1	1	−1	1	1	0	1	−1	43.84
9	1	−1	1	1	1	−1	1	−1	0	41.52
10	−1	0	−1	1	1	−1	−1	1	1	56.61
11	1	−1	−1	0	−1	1	1	1	1	66.62
12	1	1	0	−1	1	1	−1	−1	1	48.10
13	0	0	0	0	0	0	0	0	0	46.09
14	−1	−1	1	1	−1	1	−1	−1	1	40.42
15	−1	−1	−1	−1	1	0	1	−1	1	64.35
16	1	1	1	1	−1	0	−1	1	−1	31.12
17	1	1	−1	−1	1	−1	1	1	−1	51.72
18	−1	1	−1	1	0	1	1	−1	−1	26.74
19	−1	−1	0	1	−1	−1	1	1	−1	42.25
20	0	−1	−1	−1	−1	−1	−1	−1	−1	44.28
21	1	−1	−1	1	1	1	−1	0	−1	39.73

**Table 3:** ANOVA data from the screening analysis of the effect of different parameters on the pHRR response value.

Source	DF	Adj SS	Adj MS	F-Value	*p*-Value
Model	7	2751.14	393.02	85.66	0.000
Linear	7	2751.14	393.02	85.66	0.000
Specific heat capacity	1	81.58	81.58	17.78	0.001
Emissivity	1	31.07	31.07	6.77	0.022
Absorption coefficient	1	425.64	425.64	92.77	0.000
Heat of combustion	1	1306.63	1306.63	284.79	0.000
Reference temperature	1	234.65	234.65	51.14	0.000
Pyrolysis range	1	142.58	142.58	31.08	0.000
Heat of reaction	1	528.99	528.99	115.30	0.000
Error	13	59.64	4.59		
Total	20	2810.78			

**Table 4:** Design matrix and pHRR responses for sixteen fractional factorial analysis runs.

Run	$T_{ref}$	$T_{pyro}$	$h_{r}$	$C_{p}$	ε	α	$h_{c}$	pHRR
1	−1	−1	1	1	−1	1	1	55.13
2	1	1	1	1	1	1	1	47.21
3	1	−1	−1	1	1	1	−1	45.33
4	1	1	−1	1	−1	−1	1	44.48
5	−1	−1	−1	1	1	−1	1	61.90
6	1	1	−1	−1	1	−1	−1	36.16
7	1	−1	1	1	−1	−1	−1	24.88
8	−1	1	1	−1	−1	−1	1	46.32
9	1	−1	−1	−1	−1	1	1	68.07
10	1	1	1	−1	−1	1	−1	32.15
11	1	−1	1	−1	1	−1	1	46.06
12	−1	1	−1	−1	1	1	1	73.41
13	−1	1	1	1	1	−1	−1	29.68
14	−1	−1	−1	−1	−1	−1	−1	44.59
15	−1	−1	1	−1	1	1	−1	45.40
16	−1	1	−1	1	−1	1	−1	42.54

## Data Availability

The data presented in this study are available on request.

## Abbreviations

ANOVA	Analysis of variance
ATR	Attenuated total reflectance
CFD	Computational Fluid Dynamics
DNS	Direct Numerical Simulation
DoE	Design of Experiments
DSC	Differential scanning calorimeter
dTG	Derivatized thermogravimetric
FDS	Fire Dynamics Simulator
FIGRA	Fire growth rate
FRP	Fibre-reinforced polymer
FTIR	Fourier Transform infrared
HRR	Heat release rate
IL	Integral length
LES	Large Eddy Simulation
ML	Mass loss
pHRR	Peak heat release rate
RANS	Reynolds-Averaged Navier–Stokes equations
RoM	Rule of Mixtures
TGA	Thermogravimetric analysis
THR	Total heat release
TKE	Turbulent kinetic energy
TR	Turbulent resolution

## References

[1] Robert D., Baez E., Setunge S. (2021). A new technology of transforming recycled glass waste to construction components. Constr. Build. Mater..

[2] Birleanu C., Pustan M., Cioaza M., Bere P., Contiu G., Dudescu M.C., Filip D. (2023). Tribo-Mechanical Investigation of Glass Fiber Reinforced Polymer Composites under Dry Conditions. Polymers.

[3] Meunders A., Schroeder B., Spearpoint M., Arnold L. Parameter Optimization and Sensitivity Analysis for Fire Spread Modelling with FDS. Proceedings of the 10th International Conference Performance-Based Codes Fire Safety Design Methods.

[4] Nguyen Q.T., Tran P., Ngo T.D., Tran P.A., Mendis P. (2014). Composites: Part B Experimental and computational investigations on fire resistance of GFRP composite for building façade. Compos. Part B.

[5] Retardancy F., Zhang C., Shuai B., Zhang X., Hu X., Zhang H., Jia Y. (2018). Polyurethane/Red Mud Composites with Flexibility, Stretchability, and Flame Retardancy for Grouting. Polymers.

[6] Yuen A.C.Y., Chen T.B.Y., Li A., De Cachinho Cordeiro I.M., Liu L., Liu H., Lo A.L.P., Chan Q.N., Yeoh G.H. (2021). Evaluating the fire risk associated with cladding panels: An overview of fire incidents, policies, and future perspective in fire standards. Fire Mater..

[7] Zhang K., Huang J., Wang Y., Li W., Nie X. (2023). Eco-Friendly Epoxy-Terminated Polyurethane-Modified Epoxy Resin with Efficient Enhancement in Toughness. Polymers.

[8] Hurley M.J., Gottuk D.T., Hall J.R., Harada K., Kuligowski E.D., Puchovsky M., Watts J.M., Wieczorek C.J. (2016). SFPE Handbook of Fire Protecton Engineering.

[9] Nguyen K.T.Q., Weerasinghe P., Mendis P., Ngo T., Barnett J. (2016). Performance of modern building façades in fire: A comprehensive review. Electron. J. Struct. Eng..

[10] Soufeiani L., Nguyen K.T.Q., White N., Foliente G., Wang H., Aye L. (2022). Fire safety performance of 3D GFRP nanocomposite as a cladding material. Fire Saf. J..

[11] Thevega T., Jayasinghe J.A.S.C., Robert D., Bandara C.S., Kandare E., Setunge S. (2022). Fire compliance of construction materials for building claddings: A critical review. Constr. Build. Mater..

[12] Kandola B.K., Myler P., Horrocks A.R., El-hadidi M., Blair D. (2008). Empirical and numerical approach for optimisation of fire and mechanical performance in fire-retardant glass-reinforced epoxy composites. Fire Saf. J..

[13] Schartel B., Hull T.R. (2007). Development of fire-retarded materials—Interpretation of cone calorimeter data. Fire Mater. Int. J..

[14] Quan Y., Zhang Z., Tanchak R.N., Wang Q. (2022). A review on cone calorimeter for assessment of flame-retarded polymer composites. J. Therm. Anal. Calorim..

[15] Nguyen K., Kim N.K., Bhattacharyya D., Mouritz A. (2022). Assessing the combustibility of claddings: A comparative study of the modified cone calorimeter method and cylindrical furnace test. Fire Mater..

[16] Nguyen Q.T., Ngo T., Tran P., Mendis P., Zobec M., Aye L. (2016). Fire performance of prefabricated modular units using organoclay/glass fibre reinforced polymer composite. Constr. Build. Mater..

[17] Ngo T.D., Nguyen Q.T. (2016). Heat release and flame propagation in prefabricated modular unit with GFRP composite facades. Building Simulation.

[18] Kim N.K., Dutta S., Bhattacharyya D. (2018). A review of flammability of natural fibre reinforced polymeric composites. Compos. Sci. Technol..

[19] Alfakhry A.A. (2020). A comparative analytical study of some external finishing (Cladding) material in terms of their ability to spread fire in multi-story building facades in iraq. Int. J. Saf. Secur. Eng..

[20] Hassan K., Hossain D., Gilvonio M., Rahnamayiezekavat P., Douglas G. (2022). Numerical Investigations on the Influencing Factors of Rapid Fire Spread of Flammable Cladding in a High-Rise Building. Fire.

[21] Dréan V., Girardin B., Guillaume E., Fateh T. (2019). Numerical simulation of the fire behaviour of facade equipped with aluminium composite material-based claddings-Model validation at large scale. Fire Mater..

[22] Guillaume E., Fateh T., Dréan V., Girardin B. (2020). Reconstruction of Grenfell Tower fire. Part 2: A numerical investigation of the fire propagation and behaviour from the initial apartment to the façade. Fire Mater..

[23] Guillaume E., Koohkan M., Dréan V., Fateh T., Girardin B. (2020). Reconstruction of Grenfell Tower fire. Part 3—Numerical simulation of the Grenfell Tower disaster: Contribution to the understanding of the fire propagation and behaviour during the vertical fire spread. Fire Mater..

[24] (1998). Method of Test for Heat and Smoke Release Rate for Materials and Products Using Oxygen Consumpton Calorimeter.

[25] Babrauskas V. (1984). Development of the Cone Calorimeter—A Bench-scale Heat Release Rate Apparatus Based on Oxygen Consumptiont. Fire Mater..

[26] (2023). Plastics—Differential Scanning Calorimetry (DSC)—Part 1: General Principles.

[27] Wang Y., Wang Q., Wen J.X., Sun J., Liew K.M. (2017). Investigation of thermal breakage and heat transfer in single, insulated and laminated glazing under fire conditions. Appl. Therm. Eng..

[28] Zhao Y., Gordon M.J., Tekeei A., Hsieh F.H., Suppes G.J. (2013). Modeling reaction kinetics of rigid polyurethane foaming process. J. Appl. Polym. Sci..

[29] (2014). Standard Test Method for Determination of Thermal Conductivity of Soil and Soft Rock by Thermal Needle Probe Procedure.

[30] (2013). Standard Practice for General Techniques for Obtaining Infrared Spectra for Qualitative Analysis.

[31] Ma Z., Li Q., Wei J., Liang C., Yang T., Wang G. (2021). Colloids and Surfaces A: Physicochemical and Engineering Aspects Effects of Al-based alloy powders on the mechanical behavior, corrosion resistance and infrared emissivity of polyurethane composite coatings. Colloids Surfaces A Physicochem. Eng. Asp..

[32] Ahmed M.M., Trouvé A. (2021). Large eddy simulation of the unstable flame structure and gas-to-liquid thermal feedback in a medium-scale methanol pool fire. Combust. Flame.

[33] Bouchmel M., Ammar A.M., Mouldi C., Ahmed O. (2013). Large Eddy Simulation of Compartment Fire with Gas Combustible. Int. J. Mech. Mechatron. Eng..

[34] Scipión D., Fedorovich E., Palmer R., Chilson P., Botnick A. Turbulence Kinetic Energy and Dissipation Rate Estimated from a Virtual Wind Profiles and Verified through Large Eddy Simulations. Proceedings of the 34th AMS Conference on Radar Meteorology.

[35] Ketabdari M.J., Saghi H. (2011). Large Eddy Simulation of Laminar and Turbulent Flow on Collocated and Staggered Grids. ISRN Mech. Eng..

[36] Maragkos G., Rauwoens P., Merci B. (2012). Application of FDS and FireFOAM in large eddy simulations of a turbulent buoyant helium plume. Combust. Sci. Technol..

[37] Zhang X., Yang M., Wang J., He Y. (2010). Effects of computational domain on numerical simulation of building fires. J. Fire Prot. Eng..

[38] Aly H.S., Saqr K.M., Eldrainy Y.A., Jaafar M.N. (2009). Can large eddy simulation (LES) predict laminar to turbulent flow transition?. Int. J. Mech. Mater. Eng..

[39] Chen T.B.Y., Yuen A.C.Y., Yeoh G.H., Timchenko V., Cheung S.C.P., Chan Q.N., Yang W., Lu H. (2018). Numerical study of fire spread using the level-set method with large eddy simulation incorporating detailed chemical kinetics gas-phase combustion model. J. Comput. Sci..

[40] Gholami M., Fard H., Hostikka S. (2023). Combustion characteristics of non-charring polymer cylinders-experimental and numerical study. Combust. Flame.

[41] Li K., Hostikka S. (2019). Embedded flame heat flux method for simulation of quasi-steady state vertical flame spread. Fire Saf. J..

[42] Pei Y., Som S., Pomraning E., Senecal P.K., Skeen S.A., Manin J., Pickett L.M. (2015). Large eddy simulation of a reacting spray flame with multiple realizations under compression ignition engine conditions. Combust. Flame.

[43] Yuen A.C.Y., Chen T.B.Y., Yeoh G.H., Yang W., Cheung S.C.P., Cook M., Yu B., Chan Q.N., Yip H.L. (2018). Establishing pyrolysis kinetics for the modelling of the flammability and burning characteristics of solid combustible materials. J. Fire Sci..

[44] Chen X., Wang F. (2020). Performance-based engineering approach to accurately determine flame propagation characteristics over exterior video cladding wall. Fire Mater..

[45] Dréan V., Girardin B., Guillaume E., Fateh T. (2019). Numerical simulation of the fire behaviour of façade equipped with aluminium composite material-based claddings—Model validation at intermediate scale. Fire Mater..

[46] McGrattan K., Hostikka S., McDermott R., Floyd J., Weinschenk C., Overhold K. (2016). Fire Dynamics Simulator User’s Guide (FDS).

